# Cost to Save a Life in Heart Failure: Health Disparity Costs Lives

**DOI:** 10.7759/cureus.10081

**Published:** 2020-08-27

**Authors:** Philip Houck, Hari Dandapantula, Donna Wilkinson

**Affiliations:** 1 Medicine/Cardiology, Texas A&M Health Sciences Center, Temple, USA; 2 Medicine/Cardiology, Baylor Scott & White Health, Temple, USA; 3 Cardiology/Nursing, Baylor Scott & White Health, Temple, USA

**Keywords:** cost, absolute mortality, drugs, devices, surgery, payment maze

## Abstract

Objective

The purpose of this paper is to assign a dollar value to life-saving medication, surgical procedures, and medical devices. The knowledge of the wide variation in the cost of drugs, surgery, and devices allows providers and patients to choose higher-valued therapies. Cost is a significant barrier to health. The current reimbursement system is complicated, representing a significant barrier to saving lives by promoting health disparity.

Background

The cost analysis of heart failure therapies is an important tool in the education of physicians, patients, and vendors of the intervention. The analysis demonstrates disparities between heart failure therapies. The cost to save a single life is calculated from annualized absolute mortality risk reduction, trial length, and estimated 10-year costs. The method allows comparisons of drugs, devices, and surgery.

Methods

The 10-year cost of drugs is 120 months times the cost of a drug/month as listed by the website GoodRX.com. The 10-year cost of surgery or device therapy was determined from a cost analysis found by a Google search of the literature. When wide ranges were reported, the mean value was selected.

1/absolute mortality risk reduction X 100 is the number needed to treat to save a life annualized for the mean length of the study. The cost to save a life can then be computed by the following formula:

Cost/life saved = (10-year cost/annualized absolute mortality risk reduction) X (100)

Results

Beta-blockers and spironolactone had the lowest cost per life saved at $13,333 and $21,818, respectively. Defibrillators are the most expensive at $6,417,856. Valsartan/sacubitril has a cost of $1,127,733. Dapagliflozin, the newest class of heart failure drug, costs $4,853,200.

Conclusions

Calculating the cost to save a life gives insight into the value of therapies and demonstrates disparities. It is a means of comparing drugs and devices. New drug therapies are costly, not affordable, and serve as a barrier to the successful treatment of heart failure.

## Introduction

Ordering life-saving medications and interventions in heart failure seems to be a simple task determined by the guidelines. The accomplishment of this goal is more difficult and requires the knowledge to order the intervention for the correct diagnosis; patient education, agreement to be treated, and compliance with the advice; and payment of the intervention by the patient, payer, or society. The goal of this paper is to assign a cost to lifesaving therapies. This analysis will help guide physicians who order interventions. The results will educate patients on the value of their therapies, which should improve compliance. Disparities in the costs of treatments may motivate vendors to reconsider their pricing analysis. The method of this analysis estimates the cost to save a life using a 10-year cost of the intervention, the number of patients needed to treat to save one life, and the mean length of the study demonstrating an absolute mortality benefit.

Background

An economic analysis of medical treatment is a complicated calculation as outlined by Mark and Hlatky in their two-part article on medical economics [1]. The article provides a glossary of terms that are used in making an analysis to justify payments. A simpler approach is necessary to compare therapies for physicians, patients, vendors, and legislators. This simple approach has many caveats, including absolute mortality, benefits/harm, and coexisting comorbidities. The mortality benefits/harm are estimated from the literature. The cost of a drug is estimated from the website Good Rx and the costs of devices and interventions are obtained from the literature. The costs of surgical procedures and devices vary widely. The selection of 10-year costs of medications is seemingly arbitrary. Ten years is chosen since surgery and devices have a limited warranty, and 10 years seems a reasonable warranty.

## Materials and methods

Mortality of therapies and comorbidity 

Tables 1-2 list cardiac central and peripheral performance parameters, therapies, and estimated annualized absolute mortality benefit/harm based on the current literature and the mean length of the trial. Table 3 lists common comorbidities associated with heart failure exacerbation, the estimated absolute mortality conferred by the presence of the comorbid condition, and the estimated mortality benefit of the intervention. The comorbidities are renal insufficiency, arrhythmia and conduction deficits, pulmonary hypertension, anemia, obstructive sleep apnea, infection, inflammation, lymphatic dysfunction, edema, ischemic heart disease, and ischemic mitral regurgitation. Tables 3 quantifies the net effect of comorbid conditions on heart failure mortality as well as the net benefit of treatment of these comorbid conditions. It emphasizes that comorbid conditions interfere with compensatory mechanisms and contribute to mortality. Multiple comorbid conditions within a single individual is not considered. Treating comorbidity can be cost-saving.

**Table 1:** Cardiac performance parameters central acting Source [22] A – ACEI/ARB, B – Beta-blockers, S – Spironolactone, CABG – Coronary bypass surgery, MVR – Mitral valve repair replacement, ( - ) denotes increases mortality, ACEI – Angiotensin-converting enzyme, ARB – Angiotensin II receptor blocker, SGLT2 – Sodium-glucose cotransporter 2

Cardiac Performance Parameter	Therapeutic Options to Improve Heart Failure	Absolute Mortality Benefit (-) Harm	Reference
Preload	Diuretics	-8%	[2]
	Nitrates	unknown	
	Ultra-filtration	-8%	[3-4]
	CPAP	-3.3%	[5-7]
	Tolvaptan	0%	[8]
	Nesiritide	0%	[9]
Afterload	Hydralazine/Nitrate	4.2%	[10-11]
	ACEI/ARB	1.3%	[12-13]
	Nesiritide	0%	[9]
	Valsartan/Sacubitril	3.2% over A, B, S	[14]
	CPAP	-3.3%	[5-7]
Compliance	Nesiritide	0%	[9]
	Valsartan/Sacubitril	3.2% over A, B, S	[14]
	Ranexa	unknown	[15]
	Spironolactone	5.5%	[13,16]
Contractility	Digoxin	0%	[17]
	Sympathomimetics	-1.5%	[18]
	Phosphodiesterase Inhibitors	-1.5%	[18]
Geometry and Synchrony	Diuretics	-8%	[2]
	Bi-V pacing	4.1%	[19]
	Surgery CABG	1%	[20]
	Surgery MVR	2.1%	[21]

**Table 2:** Peripheral acting performance parameters Source [22] A – ACEI/ARB, B – Beta Blockers, S – Spironolactone, I – Inhibitors, ACEI – Angiotensin-converting enzyme, ARB – Angiotensin II receptor blocker, SGLT2 – Sodium-glucose cotransporter 2

Cardiac Performance Parameter	Therapeutic Options to Improve Heart Failure	Absolute Mortality Benefit Harm (-)	Reference
Neuroendocrine	ACEI/ARB	1.3%	[12-13]
	Spironolactone	5.5% over A, B	[13,16]
	Beta-Blockers	3.6% over A	[13]
	Nesiritide	0% short term	[9]
	Valsartan/Sacubitril	3.2% over B, S	[14]
Properties of Blood Vessel Agents that reduce stiffness	Spironolactone	5.5% over A, B	[13,16]
	Nesiritide	0% short term	[9]
	Valsartan/Sacubitril	3.2% over B, S	[14]
	Calorie Restriction	Data limited	[23]
Lymphatic function and Inflammation	Sympathomimetics	-1.5%	[18]
	Phosphodiesterase I	-1.5%	[18]
	Digoxin	0%	[17]
	Nesiritide	0%	[9]
	Valsartan/Sacubitril	3.2% over B, S	[14]
	Lymphedema Boots	Unknown	
Kidney function SGLT2 inhibitor	Dapagliflozin	1.2%	[24]

**Table 3:** Comorbidity/mortality - the heart failure cause/solution Source [22] RV – Right ventricle; LV – Left ventricle, LBBB – Left bundle branch block, IVCD – Intraventricular conduction delay, CHF – Congestive heart failure, Bi-V – Biventricular, CPAP – Continuous positive airway pressure, IV – Intravenous, HR – Heart rate

Comorbidity	Absolute Mortality (-) Harm	Reference	Cause	Solution
Renal insufficiency % decrease per mL/m of creatinine clearance	-1%	[25]	Over diuresis	Stop diuresis
			Bladder obstruction	Bladder scan urology consult
			Neurogenic	Straight catheterization
			Males – prostrate	Greenlight vaporization
			Females – pelvic floor	Straight catheterization
			Medications	Stop offending agent
			Decreased cardiac output	Increase cardiac output
			RV failure or restrictive LV	Increase heart rate
Arrhythmia conduction	-11%	[26]	Atrial fibrillation	Rate or rhythm control
	-19.4%	[27]	Ventricular tachycardia	Treat CHF – anti-arrhythmic
			Bradycardia – heart block	Decrease blockers pacemaker
	-3.1%	[28]	LBBB/IVCD	Bi-V pacemaker/defibrillator
Pulmonary hypertension	-25.6%	[5]	Obstructive sleep apnea	CPAP
			Lung disease	Optimize medications – O2
			Diastolic dysfunction	Medications - increase HR
			Valvular dysfunction	Valvular intervention
			Pericardial disease	Medications or intervention
Anemia	-17.3%	[29]	Iron deficiency	IV iron therapy
			Inflammation	Colchicine
			Renal insufficiency	Erythropoietin therapy
			Myelodysplasia	Erythropoietin therapy
			Testosterone deficiency	Testosterone replacement
			Vitamin deficiencies	Vitamin supplement
Infection	-.8%	[30]	Flu - viral illness	Immunizations
			Bacterial Illness	Immunizations
			Myocarditis endocarditis	IVIG
Inflammation Hs-CRP	-32%		Abnormal immune response	Colchicine
Lymphatic dysfunction	Unknown		Thoracic duct injury	Lymphedema boots
			Inhibiting medications	Discontinue offending agent
			Infection tissue injury	Treat and support
Edema	-6%		Dietary salt intake	Dietary management
			Inflammation	Unknown
			Iatrogenic medications	Remove agents
			Lymphatic dysfunction	Lymphedema boots
Coronary disease	-28%		Recurrent myocardial Infarction	Surgery, colchicine
Ischemic mitral regurgitation	-20%		Annular dilation, LV geometry	Surgery
Diabetes II	-10.2%		Insulin excess, lack of exercise, excessive calories	Emplaglitizone, exercise, good nutrition

The cost to save a life is estimated by the following formula using 10-year cost, the mean length of the study to achieve the mortality reduction, and the absolute mortality risk reduction. The cost of drug therapy is obtained from the website GoodRX.com. The 10-year cost is 120 months of the price of the drug for one month. The cost of surgery and device therapy was obtained by a Google Scholar search for cost analysis. When a spread of cost was listed the mean value was calculated.

Cost/ life saved = (10-year cost/annualized absolute mortality risk reduction) X (100)

Surveillance laboratory costs for drugs are not included since these costs are routine to general health evaluations. For devices, the follow-up expenses are also included since they are not trivial. Surgical therapies may also have follow-up costs, but they are not included.

## Results

**Figure 1:**
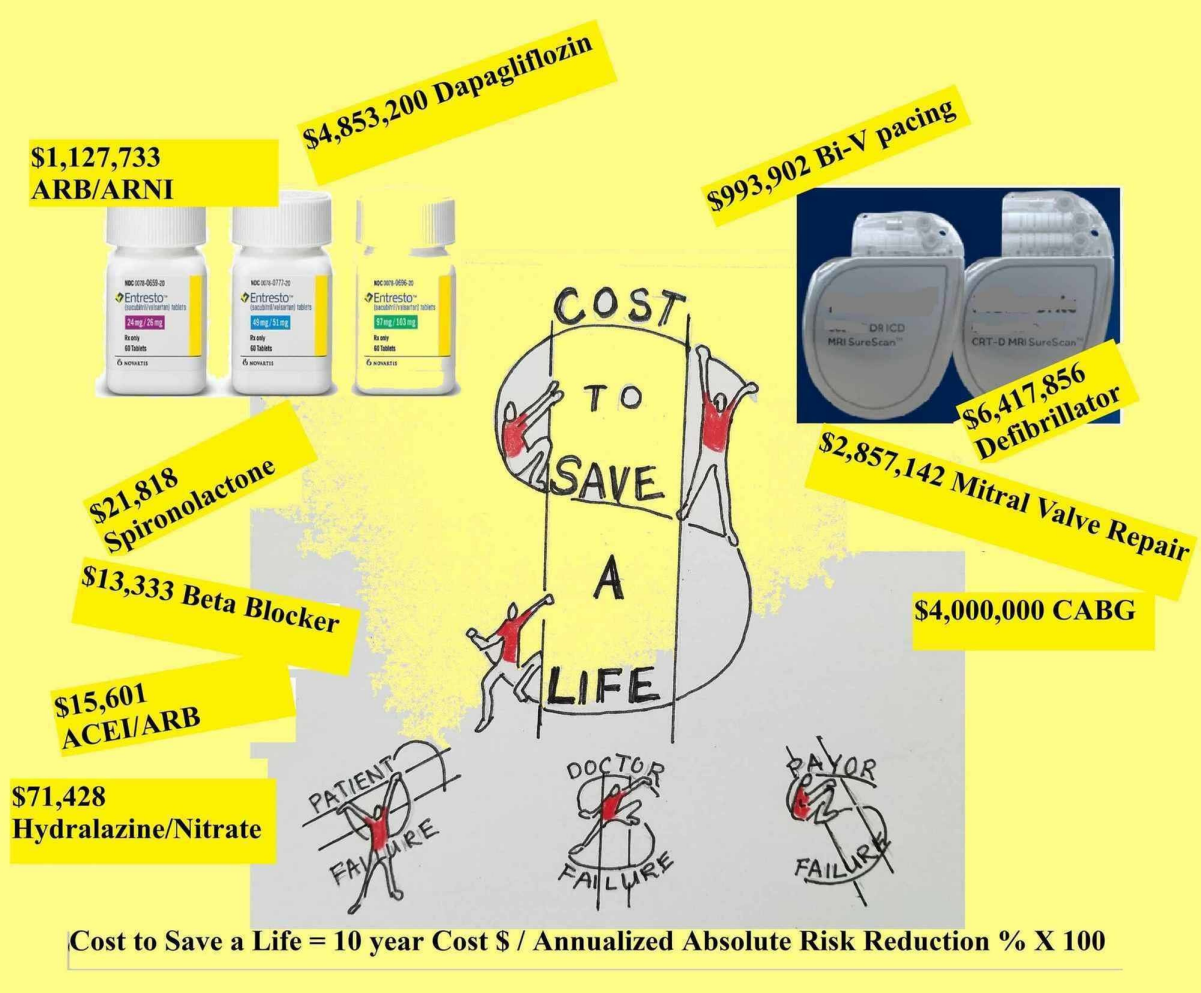
Central illustration - cost to save a life Highlights The goal of this paper is to assign a cost to lifesaving therapies. Estimating cost to save a life gives insight into the value of therapies demonstrating disparities. Beta-blockers ($13,333), Spironolactone ($21,818), Defibrillators ($6,417,856) Valsartan/Sacubitril ($1,127,733), Dapagliflozin ($4,853,200)

The authors’ estimates (based upon currently available data), summarized from Tables 1-3 of perceived benefit/harm of therapies and co-morbidities, are listed. Using annualized absolute mortality risk reduction, the number of therapies needed to treat and save a life is computed. Table 4 is useful for comparing a therapy’s mortality benefits and costs. Aldosterone inhibition appears to be a very successful strategy with a 5.5% reduction in mortality. This may be an overstatement. The cost to save a life is $21,818. A newer heart failure medication is valsartan/sacubitril with 3.2% mortality over ACEI. For fairness, the cost calculation used 4.5% absolute mortality by including the mortality benefit of ACEI at 1.3%. This is appropriate since this trial compared valsartan/sacubitril to ACEI. Even with this additional mortality benefit valsartan/sacubitril cost $1,127,733, which is nearly 50 times the cost of spironolactone and is not quite as effective. The cost of valsartan/sacubitril is comparable to mitral valve repair with coronary bypass grafting. Biventricular pacing is favored over defibrillators since it offers a better mortality benefit of 4.1% versus 1.4%. It may be better since this therapy positively remodels the heart. These devices are expensive, with defibrillators costing $6,417,856 to save a life. The newest class of drugs demonstrating a heart failure mortality benefit are the sodium-glucose cotransporter 2 (SGLT2) agents of which dapagliflozin is an example. This agent costs $4,853,200 to save a life. The 10-year cost is nearly quadruple the cost of valsartan/sacubitril. Figure 1 is a graphical summary of the results.

**Table 4:** Absolute mortality benefit and cost of life saved ACEI – Angiotensin-converting enzyme, ARB – Angiotensin II receptor blocker, CABG – Coronary bypass surgery, ICD – Implantable cardioverter-defibrillator

Medications/Interventions	Absolute Mortality Reduction %	Mean Study Follow-Up Years	10-Year Cost $	Cost/Life Saved $/Life
Spironolactone	5.5%	2	$ 1199.99	$21,818
Hydralazine/nitrate	4.2%	1.5	$ 3217.05	$71,428
Beta-blockers	3.6%	.87	$ 479.99	$13,333
Valsartan/sacubitril	3.2% over ACEI	2.1	$50748.00; includes benefit over ACEI (4.5%)	$1,127,733
ACEI/ARB	1.3%	3.45	$ 202.80	$15,601
Mitral valve repair	2.1%	4.6	$60,000	$2,857,142
CABG	1%	4.6	$40,000	$4,000,000
Biventricular pacing	4.1%	2.5	$33500+48000	$993,902
ICD	1.4%	1.7	41850+48000	$6,417,856
Dapagliflozin [24]	1.2%	1.5	$58238.40	$4,853,200

## Discussion

Mortality

Mortality is an indisputable measure. This metric depends on the population studied, length of the study, underlying disease process, immediate cause of death, and other coinciding therapies. All metrics can affect indisputable mortality.

Population studied 

Patients with the greatest mortality (New York Heart Association (NYHA) class 4, stage D) are often excluded from studies because they have high mortality. In this group, effective therapies show a significant mortality benefit as demonstrated in the spironolactone study noted below. Statisticians normalize population risk by relative mortality. The number needed to treat, however, is dependent on absolute mortality. The variability of population risk will bias the results. An example is the first vasodilator trial along with the subsequent Vasodilator-Heart Failure Trial (V-HEFT) I [23] and African-American Heart Failure Trial (A-HEFT) [10]. The population driving the success of hydralazine/nitrate therapy was a hypertensive African American population. The relative mortality benefit in the A-HEFT population was 44%, with a 4.2% absolute mortality benefit. This therapy is considered a race-specific therapy since it was only studied in African Americans. However, it is a likely therapy for those depleted in nitric oxide or over producers of free radicals [11]. The mechanism is not entirely clear. Hypertensive Caucasians, Asians, and Hispanics can have the same hypertensive genes as African Americans and could also benefit from this therapy. The 4.2% absolute mortality benefit in the A-HEFT trial may not be generalizable in a broader heart failure population since the trial selected a race-specific hypertensive population. It is equally true that failure to use this medication in hypertensive non-African Americans may exclude patients who could benefit from this therapy.

Spironolactone studied in the highest risk population appears to have the greatest mortality benefit at 5.5%, suggesting that aldosterone blockade is perhaps the most powerful heart failure strategy [16]. Aldosterone is a primitive hormonal regulator of cations and fibrosis; thus, blocking its action may be quite advantageous in preventing long-term fibrosis in a failing heart. However, spironolactone was studied in a high-risk population in the setting of co-treatment with beta-blockers and renin-angiotensin inhibitors. Thus, its benefit may be overestimated blunted by the advantage of co-therapies.

Length of the study - long-term remodeling is not assessed

Drugs used for acute exacerbations are not assessed for long-term benefits. An example is giving nesiritide only for acute exacerbation instead of continuous therapy. Continuous exposure to Brain Natriuretic Peptide (BNP) could signal repair mechanisms that positively remodel the heart over years of therapy. A mortality benefit may not be present for short duration therapy but could be significant over the long term with positive remodeling. A therapy that positively remodels the heart will have compounded benefit over a medication that simply halts decay. The first neprilysin inhibitor, valsartan/sacubitril, could have favorable structural remodeling of the heart through this action. It has not been studied in this context.

After crossing a threshold of benefit, trials are stopped. Therapies that rebuild an ailing heart may have an increasing mortality benefit over time. This benefit is not evident during the trial. Therapies that only have a short-term benefit with no positive change in the structure of the heart may appear favorable in short-duration trials but would not be expected to produce a long-term benefit. Examples are beta-blocker therapy, which induces favorable cardiac remodeling, versus left ventricular assist devices, which cause atrophy of myocardial tissue and degradation of the valves and endothelium.

Underlying disease process

Heart failure is a pleomorphic process consisting of preserved versus impaired systolic function, myofibril disease, structural heart disease, electrical system disease, inflammation, and tissue injury; thus, one size does not fit all. Cardiac abnormalities coexist with peripheral disease processes that interfere with compensatory mechanisms. The diversity of the study population can result in erroneous conclusions. Results from a highly selected population may not be successfully utilized in a general population, which may have a different disease process. An example of this concept is left bundle branch block and pacemaker-mediated cardiomyopathy that could potentially be treated with electrical therapy alone [19]. Another example is the treatment of central apnea as opposed to obstructive apnea. Central apnea is a compensatory mechanism in advanced heart failure, reducing diaphragmatic work. Treatment of this process leads to a worse outcome.

Immediate cause of death

Expert adjudication committees assign all-cause death and death due to heart failure. Death certificates are notoriously poor. A heart failure patient lacks reserve, and, in this respect, death can be attributed to heart failure. In most cases, there is an intervening condition that caused further decompensation without a substantial change in the structure of the failing heart. The specific cause of death interfered with peripheral compensatory mechanisms. Infections, anemia, falls, renal failure, obstructive uropathy, and arrhythmias are common causes of decompensation that result in a “heart failure death.” These deaths are manifestations of peripheral failure rather than a functional change in the heart’s central performance parameters.

Co-existing therapies 

Different risk populations are treated with different baseline therapies. Ethics requires providing a previously determined successful therapy. As a result, new therapies are added to old therapies, and the new mortality benefit is a measure of an add-on therapy. The new therapy is dependent on the coinciding therapies. This rationale may be faulty since interactions between therapies could lead to adverse results such as an excessive blockade of the neuroendocrine system with hyperkalemia and excessive pre-load reduction with hypotension. 

Medication errors increase when patients are hospitalized and when a greater number of medications are used. Examples include medications that interfere with creatinine clearance or interact with warfarin. More common therapies contributing to death include diuretics that appear to induce cardiorenal syndrome. Sympathomimetic/phosphodiesterase inhibitors that are used to treat cardiorenal syndrome induce arrhythmias and myocardial apoptosis. These medications are used to improve symptoms and help restore compensation at the expense of increased mortality.

The recommendations for beta-blockers, ACEI/ARB, valsartan-neprilysin inhibitors, spironolactone, defibrillators, and biventricular pacing are generally accepted. Tables 1-2 suggests that these therapies have modest improvements in survival. The greatest improvement is spironolactone, with hydralazine/nitrate in the second place (5.5% and 4.2% reductions in absolute mortality, respectively). Despite the favorable profile of these agents, they are underutilized [13]. Also, despite the known increase in mortality of the preload reduction strategy, sympathomimetic agonists, and phosphodiesterase inhibitors, these agents are over-utilized. The knowledge of mortality reduction should help Physicians improve the underutilization of spironolactone, hydralazine/nitrate. Patient education and compliance should be bolstered by hard absolute mortality numbers.

The major barrier to therapies is cost

**Figure 2:**
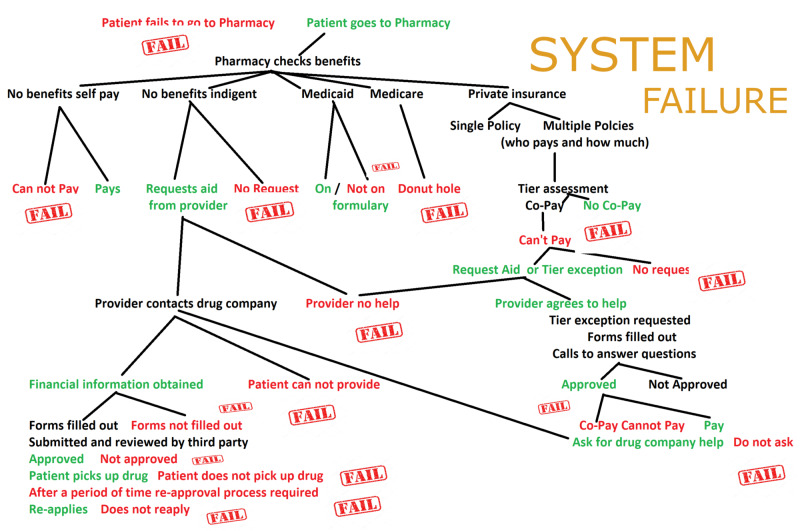
Incomplete payment maze

The third requirement for the successful treatment of heart failure is the payment of the intervention by the patient, payer, or society. Figure 2 is an attempt to summarize the maze nurses navigate to obtain patient assistance. The figure demonstrates multiple failure points preventing a patient from receiving the drug from the pharmacy. These failures are a result of a patient's and health care provider’s inability to understand the benefits, co-pays, gaps, donut holes, deductibles, and assistance that is available from drug companies. Financial forms, appeals, queries from insurance plans, tiers, tier exceptions, and, literally, multiple faxes, and peer reviews are all designed to be barriers that prevent lives from being saved.

Doctors have licensed prescriptive authority but have been usurped by third-party agents. The following letter has been useful in reminding third-party agents of the physician’s role, responsibility, and liability in caring for patients. The doctor’s orders should be completed and not negotiated. The letter is my personal communication (Houck, Philip), which I asked our hospital lawyers to draft to combat payors form usurping my prescriptive authority.

"Your pre-authorization requirement for this medication is interfering with my prescriptive authority and my ability to properly treat this patient. Your policy makes it difficult or impossible to fully observe the standard of care for treating this patient’s medical condition. When I give an order for a particular treatment, it is my intention that the order be carried out in a timely manner. Any delay in the implementation of the therapies prescribed by me could have disastrous effects on patient care. In the event that the delay created by your policy proximately causes any adverse consequences or harm to this patient, it will be my position that you should be held liable for whatever legal damages result. I urge you to withdraw your pre-authorization requirement for this medication."

Implications of non-affordable medications and devices

First, heart failure therapy is expensive. The above analysis is for single interventions. Most patients receive multiple interventions. It is not known if multiple interventions will have a greater absolute mortality benefit or just add to the cost.

Second, the cost of valsartan/sacubitril is 50 times more than that of other heart failure medications. The 10-year cost of dapagliflozin is double the cost of valsartan/sacubitril, over 4-million dollars. These agents are overpriced needlessly and are a barrier to patient treatment. The cost of 10 years of dialysis should not be equivalent to the 10-year cost of a drug. The margin on these two drugs is incredibly high versus the overhead of dialysis. The pricing of new medications should be based on margin, utilization, and value. Device therapies need to be cheaper and more precisely applied to improve the number needed to treat, lowering the cost.

Third, medical disparity, even with drug and device company financial assistance, results in failure to deliver life-saving therapies. Barriers to financial assistance need to be eliminated.

Physician leadership should take back the prescriptive authority from third-party payees. Physicians should demand a simpler method to obtain financial assistance for all patients.

## Conclusions

The simple method to estimate the cost to save a life gives insight into the value of therapies and into disparities. It is a means of comparing drugs and devices. Competition of health dollars will demand justification of costs and greater precision in delivering therapies. Physician leadership is currently lacking, with third parties usurping physician authority.
